# Scientific Education

**Published:** 1909-10-09

**Authors:** 


### Scientific Education.

The tendency which is manifested nowadays in schools to make arbitrary divisions of a "modern" or "classical" side is greatly to be deprecated. Early specialisation is more fatal than no specialisation at all, and there can be no doubt that all branches of education, if carried on in a scientific spirit, are of the utmost value in character and mind formation. Whether science as a subject will produce the effect that is secured by a classical education, as well as the result that is inseparably bound up with itself, cannot be decided definitely. It is notorious that among scientific men too little attention is often paid to the method or medium in which their views are expressed. But, on the other hand, no branch of study gives that complete satisfaction which a knowledge of properly arranged fact can bring to its possessors. Habits of accuracy and concentration are as necessary in science as in the classics, and the moral value of a scientific education is no less than that of a scientific training. So too is it the case with modern languages. If modern languages are taught with the precision and accuracy which classical teachers demand from their pupils, their use as an educating influence is invaluable. The besetting sin of schoolmasters, and its devastating consequences, have been clearly pointed out on more than one occasion, notably in an address given by Sir Clifford Allbutt some time ago. The tendency to cram the youthful mind with facts and dry formulas is destructive of mental development. The spirit of examination, as it now exists, is to find out far too severely what a man knows of the work done by other men, without ascertaining whether he appreciates the means by which their work was done. Originality is suppressed at an early date, and the result is the creation of a mass of mediocrities, who in present science echo a parrot-like cry of inefficiency. Whenever reformation is adumbrated, people are so ready to be on with the new love that they forget entirely the real merits of the old. General truths without the facts that support are almost as pernicious as facts without the truths that they illustrate. Education must combine depth with breadth, and it is better that early education should be broad rather than deep. Such a course would give a young student a basic acquaintance with the important laws of all science, before he turns his attention to the special study of one. This would prevent much of the error that is noticeable in research laboratories, where the researchers fail in their experiments because they are ignorant of the elementary principles which underlie the necessary manipulations of such an experiment.

Education therefore needs, first of all, a system, and, secondly, capable exponents of it. It will not do for the head of a school to be out of sympathy with that branch of education with which he himself is not specially concerned. Broadmindedness of the highest type is required from all teachers and a first-hand acquaintance with the subjects that they teach. It is not enough for a man to have secured first-class honours if his capacity for teaching is nil, nor should a man with fourth-class honours be rejected if he seems thoroughly interested in his subject and capable of expounding it intelligently. Too much attention is paid nowadays to academic merit. In selecting a teacher, the personal equation is the first and foremost consideration. Thus it comes about that in many cases the headmasters of schools have little more than an academic experience, and are practically without the shrewd judgment required by a man of the world. Consequently they select masters who are in most respects like themselves, and the result is that an antagonism is started between the various departments of education in a school, with necessary detriment to the pupil. The real teacher should be endowed with what might be called the spirit of statesmanship in his views on education. He should be entirely free from that bias to party glorification which mars and renders nugatory the excellence of any system.

